# C-reactive protein, haptoglobin, serum amyloid A and pig major acute phase protein response in pigs simultaneously infected with H1N1 swine influenza virus and *Pasteurella multocida*

**DOI:** 10.1186/1746-6148-9-14

**Published:** 2013-01-18

**Authors:** Małgorzata Pomorska-Mól, Iwona Markowska-Daniel, Krzysztof Kwit, Katarzyna Stępniewska, Zygmunt Pejsak

**Affiliations:** 1 Department of Swine Diseases, National Veterinary Research Institute, Partyzantów 57, Pulawy 24-100, Poland

**Keywords:** Acute phase proteins, Experimental coinfection, Swine influenza, *Pasteurella multocida*

## Abstract

**Background:**

Swine influenza (SI) is an acute respiratory disease caused by swine influenza virus (SIV). Swine influenza is generally characterized by acute onset of fever and respiratory symptoms. The most frequent complications of influenza are secondary bacterial pneumonia. The objective of this work was to study the acute phase proteins (APP) responses after coinfection of piglets with H1N1 swine influenza virus (SwH1N1) and *Pasteurella multocida* (Pm) in order to identify whether the individual APP response correlate with disease severity and whether APP could be used as markers of the health status of coinfected pigs.

**Results:**

In all coinfected pigs clinical sings, including fever, coughing and dyspnea, were seen. Viral shedding was observed from 2 to 7 dpi. The mean level of antibodies against Pm dermonecrotoxin in infected piglets increase significantly from 7 dpi. Anti-SwH1N1 antibodies in the serum were detected from 7 dpi. The concentration of C-reactive protein (CRP) increased significantly at 1 dpi as compared to control pigs, and remained significantly higher to 3 dpi. Level of serum amyloid A (SAA) was significantly higher from 2 to 3 dpi. Haptoglobin (Hp) was significantly elevated from 3 dpi to the end of study, while pig major acute phase protein (Pig-MAP) from 3 to 7 dpi. The concentrations of CRP, Hp and SAA significantly increased before specific antibodies were detected. Positive correlations were found between serum concentration of Hp and SAA and lung scores, and between clinical score and concentrations of Pig-MAP and SAA.

**Conclusions:**

The results of current study confirmed that monitoring of APP may revealed ongoing infection, and in this way may be useful in selecting clinically healthy pigs (i.e. before integration into an uninfected herd). Present results corroborated our previous findings that SAA could be a potentially useful indicator in experimental infection studies (e.g. vaccine efficiency investigations) or as a marker for disease severity, because of correlation observed between its concentration in serum and disease severity (lung scores, clinical scores).

## Background

The acute phase response is an unspecific systemic reaction of the organism that occurs after infection or inflammation [1-4]. This reaction includes changes in the concentrations of some plasma proteins called acute phase proteins (APPs) [2,5]. Changes of APP concentration in pigs serum have been extensively investigated during last years 2–4 but, to the best of our knowledge, no studies related to the APP behavior following influenza virus and *Pasteurella multocida* (Pm) coinfection has been reported.

Respiratory diseases in pigs are often considered as multifactorial problems caused by various pathogens (viral and bacterial) in combination. The most common infectious agent responsible for respiratory infection in pigs are: swine influenza virus (SIV), porcine reproductive and respiratory syndrome virus (PRRSV), *Pasteurella multocida* (Pm), *Actinobacillus pleuropneumoniae, Mycoplasma hyopneumoniae *[6,7]. These pathogens may act together to increase the severity and duration of the disease. In pigs, as well as in humans, bacterial pneumonia secondary to influenza is often observed [8] and SIV is an important contributor to the porcine respiratory disease complex (PRDC). The bacterial pathogens associated with PRDC are classified as primary or secondary pathogens, and Pm plays a key role as a secondary invader [9]. Up to now the kinetics of acute phase response after experimental infection of pigs with SIV or Pm alone have been investigated [3,4,10,11]. Exposure to multiple pathogens may result in different kinetics of APP response, as compare to monoinfection with SIV or Pm.

In this study the immune and C-reactive protein (CRP), haptoglobin (Hp), serum amyloid A (SAA) or/and pig major acute phase protein (Pig-MAP) responses after simultaneous co-infection with common porcine pathogens: SIV (H1N1 subtype) and Pm were evaluated in piglets. The correlation between concentration of investigated APP in serum and severity of infection (clinical score, lung score, turbinate score) were also studied, to estimate the utility of APP measurement in the evaluation of pigs health status.

## Results

### Clinical signs

**Figure 1:**
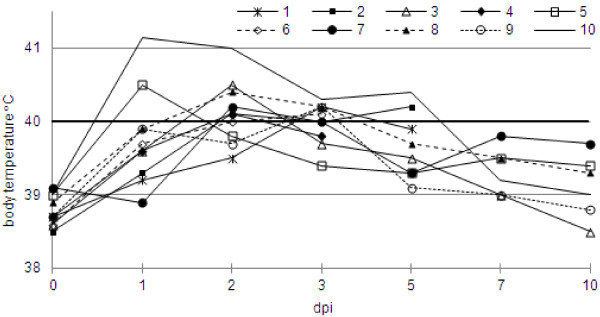
**Rectal temperature of pigs coinfected with swine influenza subtype H1N1 virus and *Pasteurella multocida*.**

In all coinfected pigs clinical sings including fever, coughing, nasal discharge, dyspnea and anorexia were observed. In all infected animals the rectal temperature increased over 40°C (Figure 1). Clinical score ranged between 1 and 5. In the control pigs no clinical signs of any disease were seen.

### Pathological examination

**Figure 2:**
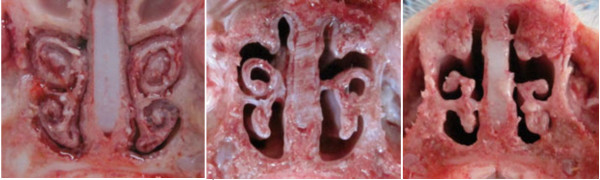
**Various degree of turbinates atrophy observed in pigs coinfected with swine influenza virus (H1N1) and *Pasteurella multocida *(A- control, B-C coinfected pigs).**

In all inoculated pigs necropsied at 10 dpi atrophy of turbinates was observed (mean TS = 1.75 ± 0.31, range (1.33-2.33) (Figure 2). In pigs euthanized at 3 or 5 dpi no clear macroscopic changes in the turbinates were observed.

**Figure 3:**
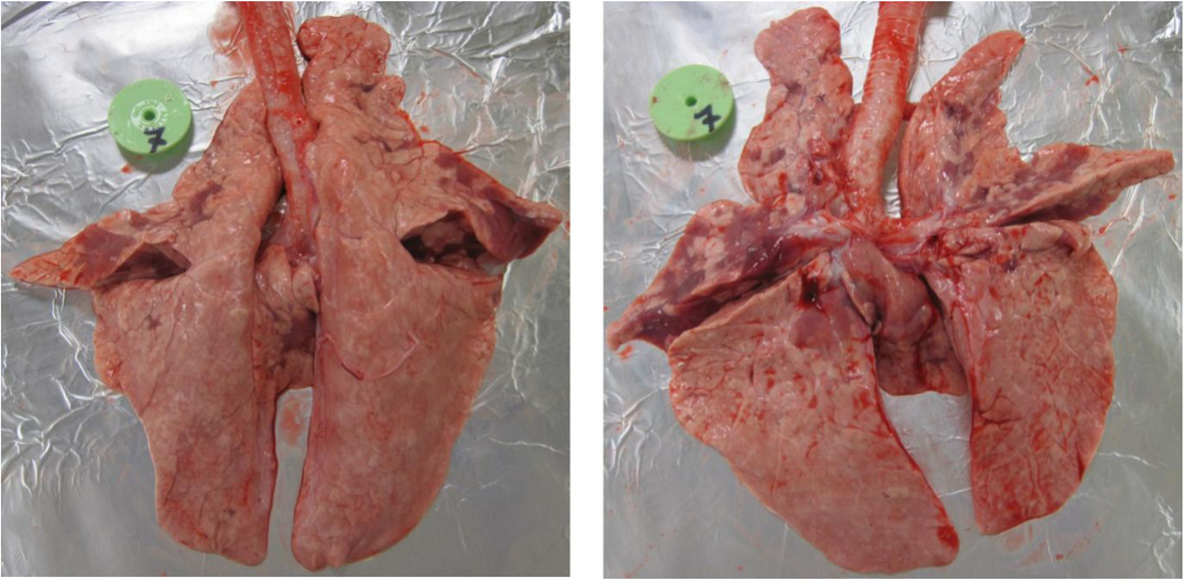
**Changes observed in the lungs of pigs coinfected with swine influenza virus (H1N1) and *Pasteurella multocida*.**

Postmortem examination revealed also macroscopic lesions in the lungs of 10/10 infected pigs. The mean lung score was 30% ± 14.93% (range 15–55%) (Figure 3). There were no significant differences between lung score observed at different days post inoculation.

### Hematological examination

**Figure 4:**
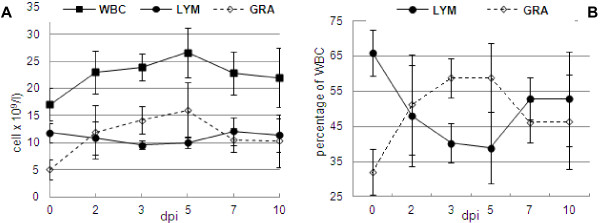
**Changes in the concentration of white blood cells, lymphocytes and granulocytes (A) and percentage of lymphocytes and granulocytes (B) in pigs coinfected with swine influenza virus (H1N1) and *Pasteurella multocida *during first 10 days after inoculation.**

In coinfected pigs, the overall number of leukocytes increased significantly during study and ranged from 17.04 × 10^9^/l at 0 dpi to 26.64 × 10^9^/l at 5 dpi (p<0.05). In control pigs total number of leukocytes ranged from 17.65 × 10^9^/l to 18.02 × 10^9^/l. The number of lymphocytes remained relatively stable, while the number of granulocytes increased significantly from 5.08 × 10^9^/l at 0 dpi to 15.77 × 10^9^/l at 5 dpi (p<0.05) and then decreased to 10.35 at 10 dpi (Figure 4A). The mean percentages of lymphocytes were the lowest at 3 and 5 dpi, and reached 40.29% and 38.86% respectively, while on day 0 the percentage of lymphocyte reached over 60% (Figure 4B). In control piglets the mean concentration as well as percentage of lymphocytes remained stable and ranged from 9.23 × 10^9^/l to 11.03 × 10^9^/l and from 53% to 67%, respectively. In infected pigs a significant increase in the percentage of granulocytes was observed from 0 to 3 and 5 dpi (from 32.84% to almost 60%) (p<0.05). Additionally, a significant increase of mean concentration of medium-sized (MID) cells, from 0.12 at 0 dpi to 0.59 × 10^9^/l, (represented mainly by monocytes) was observed at 5 dpi (p<0.05).

### Humoral immune response to SwH1N1 and Pasteurella multocida DNT

**Figure 5:**
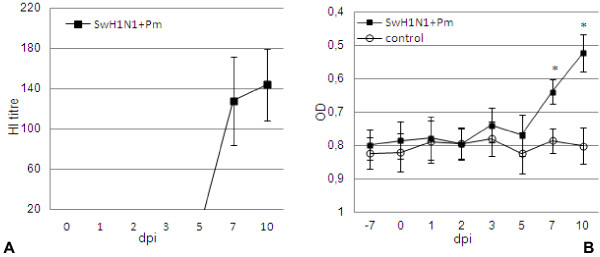
**Development in HI (A) and DNT-specific (B) antibodies in pigs coinfected with swine influenza virus (H1N1) and *Pasteurella multocida*.** Data are geometric mean ± SD. * significant increase as compared to control animals, p < 0.001.

All infected pigs exhibited specific antibodies against hemagglutinin from 7 dpi; the HI titre ranged from 80 to 160 (Figure 5A). Sera from control pigs had no antibody titres (<20 HI titre). The mean level of anti-DNT Ab started to increase significantly from 7 dpi in infected pigs as compared to controls, and tended to increase till the end of study (10 dpi) (Figure 5B).

### Presence of SIV and toxigenic Pm in swabs and tissues

Real-time RT-PCR assay, used to confirm the presence of SIV in the nasal swabs revealed positive results from all infected pigs between 2 and 5 dpi. At 7 dpi positive results were found only in 2 out of 6 infected pigs. In the nasal swabs taken before inoculation no SIV genetic material was found. In all infected pigs, euthanized at 3 and 5 dpi, the presence of SIV was confirmed for the trachea as well as middle lobes of the lungs. In three piglets, the occurrence of SIV was also confirmed for apical and accessory lobes. No viral RNA was found in diaphragmatic lobes. No viral RNA was detected in lungs on day 10 post inoculation

The results of the bacterial isolation, identification of Pm genes encoding DNT with the use of the PCR technique, and results of the real-time RT-PCR assay, used to confirm the presence of SIV in samples taken from infected pigs, are given in Table 1. In the nasal swabs taken before inoculation, no DNT producing Pm were found (bacteriological examination and PCR test). With the use of standard bacteriological method the presence of Pm was confirmed in 20 out of 30 swabs, while with the use of PCR in 26 out of 30 swabs, taken after coinfection.

**Table 1:** **Number of infected pigs (number with positive results/total number of pigs) from which the SIV and/or toxigenic *Pasteurella multocida *(Pm) were reisolated and/or identified with the use of PCR at various days after coinfection**

**dpi**	**Nasal swabs**	**Lung**
	**M gene of SIV- PCR**	
0	0/10	n/a
2	10/10	n/a
3	10/10 (++)	2/2
5	8/8 (+)	2/2
7	2/6 (+)	n/a
10	0/6	0/6
	Pm -reisolation	
0	0/10	n/a
3	6/10	0/2
5	6/8	2/2
7	4/6	n/a
10	4/6	2/6
	DNT Pm - PCR	
0	0/10	n/a
3	10/10	0/2
5	6/8	2/2
7	5/6	n/a
10	5/6	5/6

In control pigs no genetic material of toxigenic Pm and SwH1N1 were found at any time points.

### Acute phase proteins

**Figure 6:**
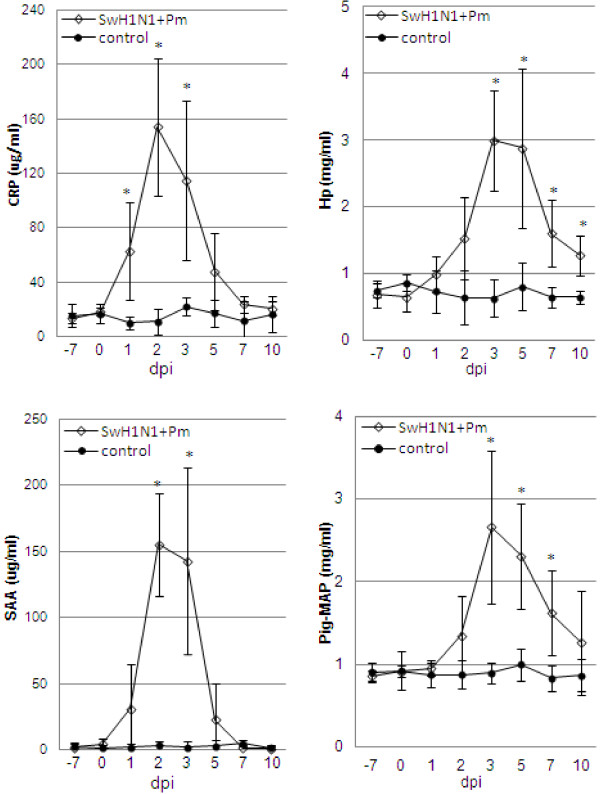
**Concentrations of CRP, Hp, SAA and Pig-MAP in serum of pigs before and at various time points after intranasal coinfection with swine influenza virus (H1N1) and *Pasteurella multocida *(mean ± SD).** *p < 0.05 – significant increase, as compared to control animals.

All investigated APP increased significantly after coinfection, with mean maximum concentration from day 2 to 3 (Figure 6). In the control pigs levels of investigated APP remained relatively constant.

#### C-reactive protein

Prior to inoculation, experimental pigs had CRP serum concentration below 22 μg/ml (mean 18.64 ± 2.59). Twenty four hour after coinfection the mean concentration of CRP reached 62.85 ± 35.55 μg/ml. Significant difference, as compared to control animals, were seen between 1 and 3 dpi (p<0.05). The maximum mean level was observed at 2 dpi and reached 153.92 ± 50.50 μg/ml (over 8-fold increase). Starting from 5 dpi after coinoculation, the mean concentrations of CRP did not differ significantly from that observed in the control pigs (p ≥ 0.05).

#### Haptoglobin

Preinoculation individual levels of Hp were found to be below 0.83 mg/ml. The highest individual level after coinfection reached 4.97 mg/ml (at 5 dpi). In all coinfected pigs the significant changes in the concentration of Hp were observed during study. The mean concentration of Hp had increased by 72 h after coinoculation and from that time-point were significantly higher as compared to control pigs (p<0.05). The highest mean concentrations of Hp were observed at 3 dpi. The mean peak level was over 4-fold higher, as compared to the mean preinoculation concentration.

#### Serum amyloid A

Significant increase of SAA after coinfection, as compared to the control pigs, was observed only at 2 and 3 dpi (p<0.05). During first 24 h after inoculation the increases of SAA concentration was not significant as compared to control pigs (p>0.05). The mean peak level reached 155.20 ± 38.93 μg/ml, this was almost 40-fold higher compared to day 0-level. From 5 dpi the SAA concentration had decreased and did not differ from those observed in control animals.

#### Pig major acute phase protein

Preinoculation levels of Pig-MAP were found to be below 0.94 mg/ml (mean 0.91 mg/ml ± 0.23). Concentration of Pig-MAP increased significantly 72 h after coinfection (p<0.05) and remained significantly elevated till 7 dpi. The highest Pig-MAP concentrations in particular pigs were detected between 3 to 5 dpi. The maximum mean concentration of Pig-MAP, observed at 3 dpi in coinfected piglets, was almost 4 times higher as compared to day 0-level. From 10 dpi the Pig-MAP concentrations had decreased and did not differ significantly between control and infected pigs.

Significant positive correlations were found between maximal concentration of Hp and SAA and lung scores (respectively r = 0.85 and r = 0.87, p<0.05). Positive correlation was also observed between maximum concentration of Pig-MAP in serum and turbinate score (r = 0.87, p<0.05) and between clinical score and Pig-MAP and SAA maximal concentrations in the serum (r = 0.87 and r = 0.85, p<0.05, respectively).

## Discussion

Swine influenza is generally characterized by acute onset of fever and respiratory symptoms [12]. The most frequent complications of influenza are secondary bacterial pneumonia. *Pasteurella multocida* is believed as a major bacterial agent that complicates swine influenza virus infections, and still remains the most common respiratory bacterium isolated in cases of PRDC [9,13]. *Pasteurella multocida* is considered to be an opportunistic invader that could be cleared from the lungs of normal pigs [13], but SIV-induced damage to the respiratory tract (loss of cilia, extrusion of mucus, exudation, necrosis and metaplasia of airway epithelium) reduced ability to clear the infection [13,14]. Previous study conducted on turkey revealed that in birds infected with avian influenza virus (AIV), the numbers of Pm in their respiratory tracts increased to a greater extent than in birds which had not been infected with the AIV [15].

Until now there have been no reports published on the kinetics of acute-phase response after coinfection of pigs with SIV and Pm, even though this coinfection is often found in the field conditions [13]. Only reports dealing with analyses of APP response in pigs monoinfected with Pm or SIV have been published to date [3,4,10,11,16].

After intranasal simultaneous coinfection of pigs with SwH1N1 and Pm various kinetics of responses could be recognized within the APP tested. The concentration of CRP increased significantly at 1 dpi as compared to control pigs, and remained significantly higher to 3 dpi. Level of SAA was significantly induced from 2 to 3 dpi. Haptoglobin was significantly elevated from 3 dpi to the end of study, while Pig-MAP from 3 to 7 dpi. The concentrations of CRP, Hp and SAA significantly increased before specific antibodies were detected.

The significant increase in the concentration of CRP, Hp, SAA and Pig-MAP have been also found previously in pigs infected with Pm only (the same strain and similar dose of Pm was used) [3]. However, in Pm monoinfection the concentration of CRP was higher, as compared to control pigs, only at 2 dpi. The mean maximum concentration of CRP in Pm-infected pigs was lower than in coinfected piglets (80.29 μg/ml and 153.92 μg/ml, respectively). In piglets infected with SIV (the same strain and similar titre of virus) the mean maximum concentration of CRP reached only 39 μg/ml [4].

Changes in Hp concentrations in present study were similar to those observed previously in Pm-infected pigs [3], but after coinfection with SIV the response were more protracted. The mean maximal concentrations were similar. In contrast in only SIV-infected pigs the level of Hp in serum increased significantly only at 1 and 2 dpi and reached considerably lower value (mean maximum concentration 1.8 mg/ml) [4].

Serum amyloid A response in coinfected pigs was observed earlier than in Pm-infected ones, but later than in SIV infected piglets [3,4]. Mean maximum concentration of SAA in serum were higher in coinfected piglets, as compared to Pm monoinfected animals (155 μg/ml and 125 μg/ml, respectively). In only SIV infected piglets the maximum level reached only 43.26 μg/ml [4]. The response of Pig-MAP in coinfected piglets were similar to those observed by us previously after single infection with Pm [3], but totally different (much intensive) from those observed after SIV infection [4,16].

Generally, in Pm and SwH1N1 coinfected pigs, the APP response was observed earlier than in Pm-infected, and the mean induction levels of most APP were higher in co-infected animals (except Pig-MAP). In comparison to SIV- infection, the APP response was much stronger and more protracted in coinfected pigs.

Interactions among multiple pathogens in pigs appear to generate a more severe or chronic outcome than is observed with individual pathogens by themselves [6,8]. Pulmonary lesions observed in present study were more severe when compared to singly infected animals [3,4]. It seems that SIV and Pm co-infection contributes to exacerbate of pulmonary lesions. There are several mechanism reported by which SIV infection predisposes to secondary bacterial infection, which include: impairment of respiratory epithelial barrier, increase host expression of receptors for bacteria leading to enhance colonization, modification of host immune responses (i.e. impairing of alveolar macrophage phagocytic function) [17-20].

Earlier response of the most APP after infection with SIV or SwH1N1 + Pm, as compared to Pm-mono infection, could be a result of shorter incubation period with regard to SIV-infections and the earliest induction of pro-inflammatory cytokines, especially IL-6, which are known to be a major regulator of APP production by hepatocytes [21]. As it have been found by Barbé et al. [11], during SIV infection IL-6 concentration in piglets’ serum peaked from 24 to 30 h post infection. At later time-points post infection, the concentration of this cytokine was either >20-fold lower or at the limit of detection. The serum levels of other cytokines stimulating production of APP by hepatocytes (IL-1, TNF-α) were under the limit of detection. Rapid disappearance of IL-6 from serum could also explain a fast drop in APP concentrations during to the preinoculation levels in only SIV infected piglets [4]. Studies conducted on mice reveled that after Pm infection concentration of cytokines involved in regulation of APP production by hepatocytes increased at later time-points post infection [22]. The concentration of IL-6 peaked at 36 h post Pm infection of mice and remained elevated for the longer period of time (at least to 96 h post infection) [22].

In the study by Francisco et al. [10], a slight correlation between Hp concentration in serum and extent of turbinate atrophy was found after infection with Pm. Our results did not confirm this finding, but we found the significant positive correlation between maximum serum Pig-MAP concentration and degree of turbinate atrophy (turbinate score). It should be mentioned that the timing of sampling must be taken into consideration as it may be critical in showing a precise correlation, as was reported previously by Francisco et al. [10]. Moreover, we found positive relationships between maximal concentration of Hp and SAA in serum and lung scores. The significant positive correlation found between maximum concentrations of SAA in serum and lung scores were also reported previously in pigs infected with H1N2 swine influenza virus [16]. Similarly, positive correlation between Hp and SAA concentration in pigs’ serum at the day of necropsy and changes in the lungs were reported after Pm infection [3]. Furthermore, the positive association between concentration of SAA and clinical score were found in present study. The positive correlation between clinical course and Hp concentration in pigs serum was also previously reported by Grau-Roma et al. [2] in pigs with postweaning multisystemic wasting syndrome.

## Conclusions

The results of our study confirmed that monitoring of APP may revealed ongoing infection, and in this way may be useful in selecting clinically healthy pigs i.e. before integration into an uninfected herd. The highest concentrations of all investigated APP were observed from 2 to 3 dpi, before specific antibodies in serum were present. Exposure to multiple pathogens resulted in the strongest CRP, Hp and SAA response as compared to mono-infection with SIV or Pm [3,4]. Additional studies need to be done in order to confirm these findings with regard to other coinfections. Present result also confirm our previous findings that SAA could be a potentially useful indicator in experimental infection studies (e.g. vaccine efficiency investigations) or as a marker for disease severity, because of correlation observed between its concentration in serum and disease severity (lung scores, clinical scores).

## Methods

### Animals

Fourteen 6-week-old piglets of the France Hybrides FH900 line were sourced from high health status herd. The herd was seronegative to porcine reproductive and respiratory syndrome virus and pseudorabies virus. No evidence of pleuropneumonia, streptococcosis and atrophic rhinitis was recorded based on clinical, serological and pathological examinations.

Prior to the start of the study all of the piglets were shown to be both influenza A virus and antibody (subtypes H1N1, H1N2, H3N2) negative by Matrix (M) gene real time RT-PCR and haemagglutination inhibition assay (HI), respectively. Animals were also tested for serum antibodies against dermonecrotic toxin (anti-DNT Ab) produced by Pm and nasal swabs from all piglets were evaluated for the presence of toxigenic Pm according to procedures described below. All piglets were shown to be anti-DNT Ab negative and no genetic material of toxigenic Pm was found.

During the experiment, piglets were housed at the BSL3 animal facility in two independent units; one for the control and one for the infected pigs. Animal use and handling protocols were approved by Local Ethical Commission.

### Preparation of inoculum

Swine influenza virus A/sw/Poland/KPR9/2004 (subtype H1N1) (hereafter referred to as SwH1N1), which had been isolated from a pig with swine influenza, was used for the experimental infection. The stock used for inoculation represented the third passage in SPF embrionated chicken eggs. The virus titer was evaluated in Madin-Darby canine kidney (MDCK) cells.

A DNT producing Pm isolate, which originated from a pig with the clinical form of progressive atrophic rhinitis, was cultured on blood agar at 37°C for 24 h. A suspension of this culture to 0.7 McFarland turbidity (which corresponds with approximately 1.5 × 10^8^ colony forming units (CFU)/ml) was prepared in PBS. A plate count was also performed to quantify the accurate number of viable bacteria (final result 1.5 × 10^8^ CFU/ml).

### Experimental design

On day 0, ten piglets were inoculated with SwH1N1 and Pm. Inoculations of 10^7.3^ TCID_50_ of SwH1N1 and 3 × 10^8^ CFU of toxigenic Pm in 4 ml of phosphate-buffered saline (PBS) were given intranasally (2 ml for each nostril). Four mock-inoculated pigs (with PBS) served as control pigs.

In order to examine the events taking place at the early stages of infection two infected and one control piglet were euthanized on days 3 and 5 after infection. The remaining pigs were euthanized and necropsied at 10 dpi.

### Clinical and pathological examination

Rectal temperatures were assessed daily and clinical signs of disease were recorded. Pigs were observed and scored for the respiratory signs as follows: respiratory rate: 0- normal, 1 – slightly elevated, 2 – moderately elevated, slight abdominal breathing, 3 – clearly elevated, distinct abdominal breathing; nasal discharge 0 – absent, 1 present; coughing 0 – absent, 1 present; sneezing 0 – absent, 1 present. All scores per topic are accumulated for a total clinical score of each individual pig (0–6). Fever was recorded when the rectal temperature was ≥ 40°C.

Blood samples were collected on −7, 0 (inoculation), 1, 2, 3, 5, 7 and 10 dpi. Nasal swabs were taken at −7, 0, 2, 3, 5, 7 and 10 dpi. Complete necropsy was done on each animal, with special emphasis on the respiratory tract. Samples from lung (all lobes separately) were collected for viral RNA and bacterial DNA extraction.

### Turbinate score

The snouts were sectioned at the upper first premolar tooth at necropsy. The lesions in the left and right turbinates and septum were scored as 0, 1, 2 and 3 as was described previously [23]. Normal turbinates were graded as 0. Slight but obvious atrophy was graded as 1. Moderate atrophy of not less than half of the turbinates was graded at 2. Severe atrophy of the dorsal and ventral scrolls was graded as 3. The three scores (from left and right turbinates and septum) were then added together and divided by 3, to determine final visual turbinate scores (TS) for each pig, ranging from 0 to 3.

### Lung score

Lungs were assessed according to the scheme described by Christensen et al. [24]. If no changes were found in the lobe, it was scored as 0%. Changes in the right lung were scored as follows: apical lobe 10%, cardiac lobe 10%, diaphragmatic lobe 35% (all together 55%). Changes observed in the left lung were scored as: apical lobe 5%, cardiac lobe 5%, diaphragmatic lobe 30%. Changes in intermediate lobe was scored as 5%. All recorded scores were then added together, to determine final visual lung score for each pig, ranging from 0 to 100%.

### Laboratory examination

#### Swabs and tissue samples

The general swine influenza A real time RT-PCR method was used for detection of SIV in swabs and tissues, as described previously [25]. Samples with Ct value <30 were considered to be M gene positive, samples having Ct value 30 to 35 with sigmoidal/logarithmic appearance were considered to be weak positive, samples with Ct value >35 were considered to be negative.

The standard bacteriological methods were used for detection of Pm in the nasal swabs and lung samples, as described previously [26]. For Pm isolation collected samples were streaked onto agar containing 5% horse blood and incubated for 24 h at 37°C in 7.5% CO_2_ atmosphere. Strains with characteristic colony morphology were identified by Api 32E tests (BioMerieux, France). Additionally, for detection of genes encoding DNT, the PCR test was performed according to the previously described procedure [27].

#### Haematological examinations

Whole blood samples were analyzed for different leukocyte proportions and concentrations on a Abacus Junior Vet 5 hematology analyzer (Diatron, Hungary). Proportions of lymphocytes, monocytes and granulocytes were calculated as a percentage of leukocyte concentration.

#### Serum analyses

Anti-DNT Ab were measured using a commercial ELISA test (PMT ELISA, Oxoid, Hampshire, UK) according to the manufacturer’s specification.

Antibodies against SIVs were measured using a haemaglutinin inhibition assay (HI) assay, performed according to the standard procedure, using 0.5% chicken erythrocytes and 4HA units of strains SwH1N1 virus. Before inoculation, to check the immune status of the piglets, the HI assay were also performed with H3N2 and H1N2 subtypes. All sera were tested in serial twofold dilutions, starting at 1:20. For estimates of the prevalence of antibodies, titres ≥ 20 were considered positive.

For determination of APP commercial ELISAs were used according to the manufacturer’s recommendation (Pig C-reactive protein ELISA and Pig haptoglobin ELISA from Life Diagnostics, Inc., USA; PigMAP KIT ELISA from PigCHAMP Pro Europa S.A, Spain; Phase Serum Amyloid A Assay from Tridelta Development Ltd County Kildare, Ireland). Serum samples were tested in duplicate. Prior to analyses samples were diluted as follows: 1:1000 for CRP, 1:35000 for Hp, 1:500 for SAA and 1:1000 for Pig-MAP.

### Statistical analysis

The obtained data were subjected to the W. Shapiro-Wilk test for normality and the Levene’s test for equality of variances. The nonparametric Friedman test was used to compare observations repeated on the same subjects. Comparisons between infected and control groups at each time point were assessed using the Mann–Whitney *U* test. For analysis of correlation the Spearman Rank correlation test was used. For all analyses, p < 0.05 was considered significant. All calculations were performed with Statistica 8.0 (Statsoft).

## Competing interests

The authors declare that they have no competing interests.

## Authors’ contributions

MPM designed the experiments and analyzed the experimental data and performed statistical analysis. KK and MPM performed the experiments (inoculation, necropsy clinical examination), prepared the serum and tissue samples, laboratory examination. KS helped to perform the experiments (bacteriology). MPM, IMD and ZP prepared the manuscript and supervised the experiment. All authors have read and approved the final manuscript.
